# Supplementary material for: An assessment of China’s methane mitigation potential and costs and uncertainties through 2060
Source: Nat Commun. 2024 Nov 8;15:9694. doi: 10.1038/s41467-024-54038-y (PMC11549391; doi:10.1038/s41467-024-54038-y)
Supplement: Supplementary file 1 — Supplementary Information [file 41467_2024_54038_MOESM1_ESM.pdf]

An Assessment of China's Methane Mitigation Potential and Costs and Uncertainties through 2060

Nina Khanna[1], Jiang Lin[1,2]*, Xu Liu[3], and Wenjun Wang[2]

Affiliations: [1] Energy Technologies Area, Lawrence Berkeley National Laboratory; Berkeley, CA 94720, USA; [2] University of California at Berkeley; Berkeley, CA 94720, USA; [3] Peking University, Beijing, China.

*Corresponding author. Email: j_lin@lbl.gov

Supplementary Information

Supplementary Table 1. China's Reported and Calculated Methane Emissions from This Study

Supplementary Table 2. China's Methane Emission Source Activity Projections under All Scenarios

Supplementary Table 3. Key assumptions for methane mitigation measures, methane reduction rates and cost estimates under Cost-effective and Deep Mitigation Scenarios

Supplementary Table 1. China's Reported and Calculated Methane Emissions from This Study

		**2014 CH4 Emissions (100-yr GWP, $MtCO_2e$)**	**2017 CH4 Emissions (100-yr GWP, $MtCO_2e$)**
Energy Sector	National Communications' GHG Inventory	520	860
	This Study's Results	631	804
Agriculture	National Communications' GHG Inventory	467	741
	This Study's Results	424	738
Waste and Wastewater	National Communications' GHG Inventory	138	220
	This Study's Results	155	240
Total	National Communications' GHG Inventory	1125	1821
	This Study's Results	1210	1782

Source: 2014 GHG Inventory data from MEE, 2019[1]; 2017 GHG Inventory data from MEE, 2023[2].

Note: CH4 emissions converted from physical units (kt) reported in China's National Communication reports to $MtCO_2e$ using IPCC AR5 100-yr GWP values for comparability to our study's results.

Supplementary Table 2. China's Methane Emission Source Activity Projections under All Scenarios

		Reference and Cost-Effective Mitigation Scenarios				Deep Mitigation Scenario			
		2020	2030	2050	2060	2020	2030	2050	2060
**Energy**									
Coal production	Mtce	2762	2170	701	638	2762	2170	420	383
Abandoned coal mine capacity	Mtce	130	182	84	36	130	182	51	22
Crude oil production*	Mtce	328	300	171	63	328	300	171	63
Natural gas production*	Mtce	137	62	10	10	137	62	10	10
Natural gas transmission & distribution	Mtce	257	357	424	411	257	291	147	142
Biomass combustion	Mtce	132	128	79	58	132	78	35	21
Transportation diesel and gasoline consumption	Mtce	6825	7717	4963	2481	6825	4898	0	0
**Agriculture**									
Rice cultivation total harvest area (4 growth types)	million ha	32	31	31	31	Same as Reference and Cost-Effective Mitigation scenarios			
Livestock									
Sheep and Goats	million	364	389	361	332				
Hogs	million	474	490	452	419				
Camels	million	0.4	0.4	0.4	0.4				
Mules	million	1	1	1	1				
Donkeys	million	4	3	3	3				
Horses	million	5	4	4	4				
Cattle and buffalo	million	121	126	118	108				
Freshwater aquaculture Production	Mt	36	46	43	40				
**Waste and Wastewater**									
Solid Waste	Mt	171	169	134	97				
Industrial Wastewater COD	Mt	33	42	53	58				

Domestic Wastewater BOD	Mt	21	21	19	18	

Note: Mt is million metric tons, kt is thousand metric tons, ha is hectares, kg/cap is kilograms per capita, and Mtce is million metric tons of coal equivalent, the standard unit for energy used in Chinese statistics and equal to 29.27 million gigajoules. COD is chemical oxygen demand and BOD is biochemical oxygen demand.

* Energy sector activity projections vary by scenario due to two different paces of clean energy transition assumed. China's oil and gas production capacity remains the same under both scenarios as it is heavily import dependent, so reductions in demand are reflected in imports and not domestic production. Transmission and distribution reflect movement of domestically produced oil and gas as well as imported oil and gas to meet total demand.

Supplementary Table 3. Key assumptions for methane mitigation measures, methane reduction rates and cost estimates under Cost-effective and Deep Mitigation Scenarios

Sector and GHG Targeted	Mitigation Measure	Details of Mitigation Measure	Cost-Effective Mitigation Scenario Assumptions	Deep Mitigation Scenario Assumptions	Cost Estimates or Other Cost-related Information
Energy Sector					
Coal Mining	Ventilation air methane (VAM) oxidation<br><br>Gas collection for flaring and/or for energy use	VAM oxidation combusts low-concentration methane into H_2O and CO_2.<br><br>Drainage and flare systems recover high-concentration fugitive coal mine methane gas from underground coal seam that can be used for electricity generation.	Assume linear growth in methane reduction efficiency of VAM oxidation and recovery from 0% in 2020 to 38% by 2050, and reduction efficiency for flaring and energy use to 28% by 2025, and constant thereafter [EPA[3], Yang et al.[4]].	Assume linear growth in methane reduction efficiency of VAM oxidation and recovery from 0% in 2020 to 38% by 2025, and reduction efficiency for flaring and energy use to 28% by 2025, and constant thereafter.	Used average cost from literature range of US$5.42 - $29.2/$tCO_2e$ for a combination of both measures [Yang et al.[4], Höglund-Isaksson[5], and EPA[6]]
Abandoned Coal Mine Methane	None, but activity changes in abandoned coal mine capacity affects methane emissions	No direct mitigation measures considered due to lack of data on mitigation applicability, costs, and technological immaturity of mitigation measures [Kang et al.[7]]	More gradual phasedown of coal as a share of total energy consumption, resulting in more abandoned coal mine capacity in later years with 64 Mt of abandoned capacity annually in 2060.	Faster phasedown of coal as a share of total energy consumption, resulting in greater abandoned coal mine capacity earlier and 42 Mt of abandoned capacity annually in 2060.	Costs not considered for indirect changes in activity levels
Oil Production, Transmission & Distribution	Installing vapor recovery units, directed inspection and maintenance, and good practices<br><br>Recovery and utilization of		None due to individual measure cost that all exceed the $10/$tCO_2e$ threshold for this scenario	Assume linear growth in methane reduction efficiency of combined measures from 0% in 2020 to 24% (i.e., 13% from inspection, maintenance and good practices; 11% from recovery and utilization of vented gas)	Combined mitigation costs of about $40/$tCO_2e$ in 2030 based on mitigation measures' costs from literature [EPA[6]; Höglund-Isaksson[5]]

	vented gas during production			in 2030. [EPA[6]; Höglund-Isaksson[5]]	
Natural Gas Production, Transmission & Distribution	Green completions, plunger lift systems, leak monitoring and repair (LDAR), and low- or no-bleed or air pneumatic controllers	Green completions use portable equipment to capture fugitive methane during well completion.<br><br>Plunger lift systems lift a column of accumulated fluid out of a well to replace beam lifts and well blowdowns (i.e., venting)[8].<br><br>Using low- or no-bleed (i.e., low or no venting) or air pneumatic controllers can reduce methane leakage[3,9-10]	Assume emissions reduction efficiency increases linearly to reach its maximum combined reduction rate of 52% by 2025 based on applicability in China and remains constant thereafter [EPA[3], Brink et al.[9], NRDC[10]].	Same as Cost-effective Mitigation Scenario	Weighted average costs of $8/tCO_2e$ in 2030 based on mitigation costs found in EPA[6] ; IEA[11]; and Höglund-Isaksson[5]
Biomass Combustion	Indirect mitigation through fuel switching for rural heating and cooking	Technology fuel switching (e.g., from biomass-based heating and cooking to electric heating and cooking)	Biomass share of rural residential heating varies by climate zone, but declines to 25% in South region and from 45% in 2020 to 40% in 2060 in Transition region and are replaced by electric end-uses. Biomass share of rural cooking remains relatively constant at 28% in 2020 and 30% in 2060 [updated analysis based on Zhou et al.[12]].	Biomass share of rural residential heating varies by climate zone, but declines to 0% in South region and from 45% in 2020 to 20% in 2060 in Transition region. Biomass share of rural cooking decreases from 28% in 2020 to 0% in 2060. Heating and cooking are replaced by electric end-uses [updated analysis based on Zhou et al.[12]].	Costs not considered for indirect mitigation
Transport Diesel &	Indirect mitigation through vehicle electrification	Vehicle technology fuel switching (e.g., from diesel and gasoline	Over 50% of private car, bus and light-duty fleet is electrified by 2060,	100% electrification of passenger road fleet and light- and medium-duty	Costs not considered for indirect mitigation

Gasoline Combustion		vehicles to electric vehicles)	replacing gasoline and diesel vehicles [updated analysis based on Zhou et al.[12]].	trucks, and over 80% electrification of heavy-duty trucks by 2060. [updated analysis based on Zhou et al.[12]].	
Agriculture					
Rice Cultivation	Changing irrigation method<br><br>Biochar application	Examples include alternate wetting and drying irrigation, intermittently aerated and continuously flooded irrigation methods.<br><br>Application of biochar – partially combusted biomass – as a soil supplement to absorb methane.	Methane reduction efficiency for irrigation method changes increases linearly to 7% in 2020 and constant thereafter, and biochar reduction efficiency increases linearly to 26% in 2030 and constant thereafter based on Chinese expert interviews, EPA[6] and Höglund-Isaksson[5] for cost-effectiveness of irrigation methods and Sun[13], Mohammadi et al.[14], and Clare et al.[15] for biochar applicability.	Methane reduction efficiency for irrigation method changes increases linearly to 31% in 2030 and constant thereafter, and biochar reduction efficiency increases linearly to 26% in 2030 and constant thereafter based on Chinese expert interviews, EPA[6] and Höglund-Isaksson[5] for costs of irrigation methods and Mohammadi et al.[14] and Clare et al.[15] for biochar applicability.	Rice cultivation irrigation methods: costs range from alternate wetting costs of \$16.17/tCO_2e to combination of methods that costs \$25-\$32/tCO_2e [EPA[6]; Höglund-Isaksson[5]]<br><br>Biochar: average of \$14/tCO_2e from literature on China and Vietnam [Mohammadi et al.[14], Clare et al.[15]]
Enteric Fermentation	Improving nutritional balance and feed conversion of livestock feed, and intensive grazing	Improving nutritional balance of livestock feed to improve feed digestibility and conversion efficiency with increased rate of production from livestock, and managed intensive grazing to reduce methane emissions	Assume linear growth of emission reduction efficiency to 10% in 2025 based on Chinese expert interviews and EPA[6].	Same as Cost-Effective Scenario. See discussion of Enteric Fermentation sensitivity scenario for additional emerging mitigation measures.	Estimated average cost of US\$63/tCO_2e based on costs data from EPA[6] and Höglund-Isaksson[5].
Manure Management	Converting manure to compost and anaerobic digesters	Dry manure can be managed by converting	Assume linear growth of combined emission reduction efficiency to 50% in 2025, and remaining	Same as Cost-Effective Mitigation Scenario	Costs not included in cost-curve analysis due to significant variations between different

		it to compost production.<br><br>Anaerobic digesters use microorganisms to breakdown livestock manure and produces biogas that can be captured and used as heat or electricity.	constant thereafter, based on Chinese expert interviews, EPA[6], and Höglund-Isaksson[5].		studies, ranging from \$36/tCO_2e–\$47/tCO_2e for digesters from Höglund-Isaksson[5] and combination of measures with costs ranging from \$7/tCO_2e–\$98/tCO_2e in 2030 in EPA[6].
Aquaculture	Switching from extensive and semi-intensive to intensive systems	Switching from shallow, extensive systems to intensive and continuously aerated systems can result in higher fish production and reduced methane emissions	Assume linear growth of methane reduction efficiency to reach 100% intensive systems by 2035 due to net positive economic benefits.	Same as Cost-Effective Mitigation Scenario.	Net cost of -\$128/tCO_2e, when considering the sum of production costs and gross revenue from additional yield, based on China-specific costs from Yuan[16] and Yuan et al.[17]
Waste					
Solid Waste	Collection and flaring and capturing landfill gas for energy use	Collect and directly flare methane released from landfills or use for electricity generation	Assume linear growth of methane reduction efficiency to reach 35% by 2025 and remain constant thereafter, based on EPA[3] and Yang et al.[4]	Same as Cost-Effective Mitigation Scenario.	Estimated averaged cost of \$7/tCO_2e based on China-specific costs reported in EPA[3], Yang et al.[4] and Höglund-Isaksson[5]
Wastewater					
Domestic Wastewater	Anaerobic and aerobic wastewater treatment	Upgrade to anaerobic sludge digesters with gas recovery and utilization; and changing open sewer, latrines and septic tanks to aerobic wastewater treatment plant (WWTP)	None due to individual measure cost that all exceed the \$10/tCO_2e threshold for this scenario	Assumes linear growth in methane reduction efficiency to 11% by 2030 and constant thereafter based on EPA[6].	Averaged costs from existing literature for China to \$24/tCO_2e [EPA[6], Höglund-Isaksson[5]]
Industrial Wastewater	Anaerobic and aerobic	Upgrade to anaerobic sludge digesters with	None due to individual measure cost that all	Assumes linear growth in methane reduction efficiency	Averaged costs from existing literature for

	wastewater treatment	gas recovery and utilization; and changing open sewer, latrines and septic tanks to aerobic wastewater treatment plant (WWTP)	exceed the $10/tCO$_2$e threshold for this scenario	to 11% by 2030 and constant thereafter based on EPA[6] .	China to $24/tCO$_2$e [EPA[6], Höglund-Isaksson[5]]

Note: The baseline Reference Scenario for assessing methane mitigation potential from Cost-Effective and Deep Mitigation scenarios do not consider any methane mitigation actions.

Supplementary References:

1. Ministry of Ecology and Environment (MEE) of the People's Republic of China. *The People's Republic of China Second Biennial Update Report on Climate Change* (MEE, Beijing, 2019). http://qhs.mee.gov.cn/kzwsqtpf/201907/P020190701765971866571.pdf

2. MEE. 2023. *The People's Republic of China Fourth National Communication on Climate Change.* (MEE, Beijing, 2023). https://unfccc.int/documents/636695

3. U.S. EPA. *Global Mitigation of Non-CO_2 Greenhouse Gas Emissions: 2010–2030.* (U.S. EPA, Washington, DC, 2013). https://www.epa.gov/sites/default/files/2016-06/documents/mac_report_2013.pdf.

4. Yang, L., Zhu, T. & Gao, Q. *Technologies and Policy Recommendations for Emission Reduction of Non-CO_2 Greenhouse Gas from Typical Industries in China (in Chinese)* (China Environment Press, Beijing, 2014).

5. Höglund-Isaksson, L. Global anthropogenic methane emissions 2005–2030: Technical mitigation potentials and costs. *Atmos. Chem. Phys.* **12**, 9079–9096 (2012).

6. U.S. Environmental Protection Agency (EPA) *Global Non-CO_2 Greenhouse Gas Emission Projections & Mitigation Potential: 2015-2050*. (U.S. EPA, Washington, DC, 2019).

7. Kang, Y., Tian, P., Li, J., Wang, H. & Feng, K. Methane mitigation potentials and related costs of China's coal mines. *Fundamental Res.*, In Press. https://doi.org/10.1016/j.fmre.2023.09.012 (2023)

8. United States Environmental Protection Agency (EPA). *Installing Plunger Lift Systems in Gas Wells* (U.S. EPA, Washington, DC, 2016). https://www.epa.gov/sites/production/files/2016-06/documents/ll_plungerlift.pdf

9. Brink, S., Godfrey, H., Kang, M., Lyser, S., Majkut, J., Mignotte, S. Methane Mitigation Opportunities in China. (Princeton University, Princeton, 2013). https://www.princeton.edu/~mauzeral/teaching/WWS591e_Methane_Workshop_FinalReport%202013.pdf.

10. Natural Resources Defense Council (NRDC). Controlling Methane Emissions from the Natural Gas Systems (in Chinese) (NRDC, Beijing, 2012). http://www.nrdc.cn/Public/uploads/2016-12-04/5843d6a697a95.pdf

11. IEA. *Greenhouse gas emissions from energy*. (IEA, Paris, 2021) https://www.iea.org/data-and-statistics/data-product/greenhouse-gas-emissions-from-energy

12. Zhou, N., et al. China Energy Outlook 2022. (Lawrence Berkeley National laboratory, Berkeley, 2022). https://international.lbl.gov/sites/default/files/2022-04/China%20Energy%20Outlook%202022-full%20report%2004.22.22.pdf

13. Sun, X.; Ding, J.; Jiang, Z.; Xu, J. Biochar improved rice yield and mitigated CH4 and N2O emissions from paddy field under controlled irrigation in the Taihu Lake Region of China. *Atmos. Environ*. **200**, 69-77 (2019).

14. Mohammadi, A.; Cowie, A.L.; Cacho, O.; Kristiansen, P.; Anh Mai, T.L.; Joseph, S. Biochar addition in rice farming systems: Economic and energy benefits. *Energy* **140**, 415–425 (2017).

15. Clare A, Shackley S, Joseph S, Hammond J, Pan G, Bloom A. Competing uses for China's straw: the economic and carbon abatement potential of biochar. *GCB Bioenergy* **7**, 1272e82 (2015).

16. Yuan, X. 2007. *Economics of aquaculture feeding practices: China*. In M.R. Hasan (ed.). Economics of aquaculture feeding practices in selected Asian countries. (FAO, Rome, 2007).

17. Yuan, J., et al. Rapid growth in greenhouse gas emissions from the adoption of industrial-scale aquaculture. *Nat. Clim. Change* **9**, 318-322 (2019).
